# Mechanical ventilation and the daily cost of ICU care

**DOI:** 10.1186/s12913-020-05133-5

**Published:** 2020-03-31

**Authors:** Klaus Kaier, Thomas Heister, Jan Wolff, Martin Wolkewitz

**Affiliations:** 1 Institute for Medical Biometry and Statistics, Faculty of Medicine and Medical Center, University of Freiburg, Stefan-Meier-Straße 26, 79104 Freiburg, Germany; 2 Klinik für Psychiatrie und Psychotherapie Universitätsklinikum Freiburg Medizinische Fakultät Albert-Ludwigs-Universität Freiburg, Freiburg, Germany; 3 Evangelische Stiftung Neuerkerode, Klostergang 66, 38104 Braunschweig, Germany

**Keywords:** Mechanical ventilation, Critical care, Daily cost, ICD-10

## Abstract

**Background:**

Intensive care units represent one of the largest clinical cost centers in hospitals. Mechanical ventilation accounts for a significant share of this cost. There is a relative dearth of information quantifying the impact of ventilation on daily ICU cost. We thus determine daily costs of ICU care, incremental cost of mechanical ventilation per ICU day, and further differentiate cost by underlying diseases.

**Methods:**

Total ICU costs, length of ICU stay, and duration of mechanical ventilation of all 10,637 adult patients treated in ICUs at a German hospital in 2013 were analyzed for never-ventilated patients (*N* = 9181), patients ventilated at least 1 day, (*N* = 1455) and all patients (*N* = 10,637). Total ICU costs were regressed on the number of ICU days. Finally, costs were analyzed separately by ICD-10 chapter of main diagnosis.

**Results:**

Daily non-ventilated costs were €999 (95%CI €924 - €1074), and ventilated costs were €1590 (95%CI €1524 - €1657), a 59% increase. Costs per non-ventilated ICU day differed substantially and were lowest for endocrine, nutritional or metabolic diseases (€844), and highest for musculoskeletal diseases (€1357). Costs per ventilated ICU day were lowest for diseases of the circulatory system (€1439) and highest for cancer patients (€1594). The relative cost increase due to ventilation was highest for diseases of the respiratory system (94%) and even non-systematic for patients with musculoskeletal diseases (13%, *p* = 0.634).

**Conclusions:**

Results show substantial variability of ICU costs for different underlying diseases and underline mechanical ventilation as an important driver of ICU costs.

## Background

Intensive care units (ICUs) represent one of the largest clinical cost centers in hospitals [1]. Mechanical ventilation (MV) accounts for a significant share of this cost [2]. Patients requiring MV represent a substantial share of all ICU patients and have been shown to account for a disproportionately high share of total ICU costs [2–5]. Intensive care patients require therapy that varies considerably in type, duration, and cost. Despite the need for detailed cost data to inform policy makers, there is a relative dearth of information relating to the daily cost of ICU care for different patient groups and the impact of MV on these costs [2]. Therefore, the objectives of this analysis is to determine the daily costs of ICU care, the incremental cost of MV per ICU day, and to further differentiate these cost figures for patients with different underlying diseases. This, we argue, is an important step towards a better understanding of resource utilization in hospitals and quantifying the burden of diseases requiring intensive care treatment.

## Methods

We collected total ICU costs, length of ICU stay, and duration of mechanical ventilation of all 10,637 patients aged 18 years or older treated in ICUs at the University Medical Center Freiburg (UMCF) in 2013. The UMCF is a tertiary care teaching hospital in southern Germany.

Cost figures were calculated using the widely used bottom up microcosting approach according to the standardized methods of the Institute for the Payment system in Hospitals (InEK), which is the German calculation authority responsible for reimbursement rates [6–8]. This de facto accounting standard not only allows the calculation of reimbursement rates for each G-DRG, but it is also suitable for strategic planning and benchmarking, and, due to its accuracy and transparency, for cost analysis [7]. Briefly, according to the InEK handbook of calculation the cost object accounting is based on a defined cost template and corresponding cost categories [9]. Costs are divided into three main categories: [1] staff costs, [2] material costs and [3] infrastructure costs. Within the three categories, a total of 11 different cost centers are calculated (see Additional file 1). Cost allocation on each inpatient case generates a uniform cost-matrix and relies on a full cost approach using real costs. Direct costs, which are mandatory for implants, blood products or drugs etc., are based on the documented utilization. Overhead costs and costs on primary cost units are charged based upon key cost drivers. Amounts for indirect cost units such as on demand medication or dressing material are allocated to primary cost units and are excluded if they are not relevant for the corresponding G-DRG [7, 9]. Labor costs, which are crucial in ICU settings, are measured according to actual utilization of the respective caregiver (medical staff, nursing staff, and technical staff; see Additional file 1). Daily ICU costs were not available for analysis. Instead, we analyzed total ICU costs within three different groups of ICU patients: never-ventilated ICU patients (*N* = 9181), ICU patients ventilated at least 1 day, (*N* = 1455) and all ICU patients (*N* = 10,637). We used linear regression models, regressing total ICU costs on the number of ICU days. For the second and third group we additionally regressed on the number of ventilation days. This way we estimate and compare the daily costs of ICU stay between the three groups in order to determine whether estimated costs of a single non-ventilated ICU day are different between never-ventilated patients and patients that are ventilated at some point in their ICU stay. Finally, we differentiated our estimation for the patients’ underlying diseases by repeating the above described analyses for subgroups of patients of each ICD-10 chapter separately, using the patients’ main diagnosis (the main reason for hospitalization). All results regarding the impact of ventilation on daily ICU cost were shown on an absolute and relative scale. For all statistical analyses Stata Version 15.1 (Stata Corp, College Station, Texas, USA) is used.

## Results

**Fig. 1:**
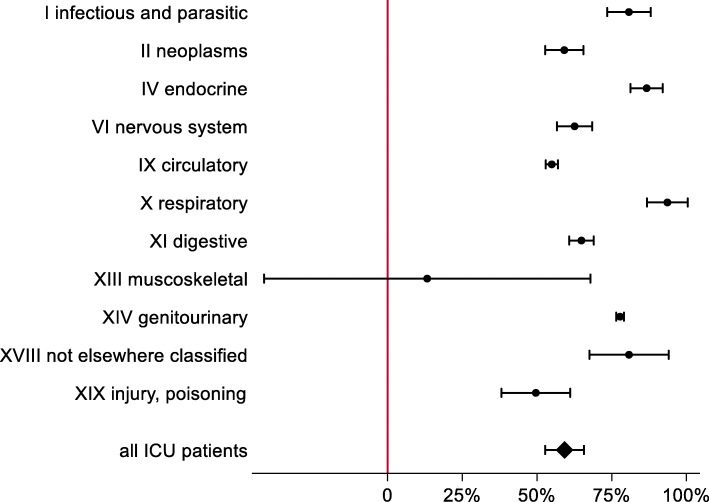
Relative increase in daily costs due to mechanical ventilation. Note: Subgroups of patients with identical ICD-10 chapters but *N* < 100 were not analyzed. In subgroups where less than 2 patients were ventilated, costs per ventilated ICU day are not shown. Figure created with Microsoft Office

In 2013, 10,636 patients were treated in ICUs at UMCF. Of these patients, a total of 1455 (14%) received mechanical ventilation. As shown in Table 1, daily costs of non-ventilated ICU care were €999 (95%CI €924 - €1074), and daily costs per ventilated ICU day were €1590 (95%CI €1524 - €1657). The cost per non-ventilated ICU day is similar between those never-ventilated and patients ventilated at some point during their hospital stay. This suggests that the higher total daily costs of ventilated patients may be attributed to the costs of ventilation rather than the underlying disease. While the higher costs of mechanical ventilation can in part be a reflection of the costs calculation method, one would expect the underlying disease to be responsible for a large part of the cost. For different underlying diseases the share of patients receiving mechanical ventilation varies widely: 41% of all ICU patients with infectious or parasitic diseases (ICD-10 Chapter I) were ventilated, but (almost) no patients with eye or ear related diseases or pregnancy were ventilated (see Table 1). Estimated costs per ventilated ICU day were lowest for patients with diseases of the circulatory system (€1439) and highest for cancer patients (€1594). In contrast, costs per non-ventilated ICU day differed substantially and were lowest for patients with endocrine, nutritional or metabolic diseases (€844), and highest for patients with musculoskeletal diseases (€1357). Average cost increase due to mechanical ventilation was 59% (95%CI 53–66%) (Fig. 1). The cost increase due to ventilation was highest for patients hospitalized due to diseases of the respiratory system (94%), and even non-systematic for patients with musculoskeletal diseases (13%, *p* = 0.634).

**Table 1:** Descriptive statistics

	Cost per non-ventilated ICU day [95%CI]	Cost per ventilated ICU day [95%CI]	Number of patients that were ventilated, N,%
**Never-ventilated ICU patients**	961 €		0, 0%
***N*** **= 9181**	[926 € - 993 €]		
**Ventilated ICU patients**	1062 €	1617 €	1455, 100%
***N*** **= 1455**	[870 € - 1253 €]	[1504 € - 1730 €]	
**All ICU patients**	999 €	1590 €	1455, 14%
***N*** **= 10,636**	[924 € - 1074 €]	[1524 € - 1657 €]	
**I infectious and parasitic**	864 €	1561 €	98, 41%
***N*** **= 239**	[833 € - 894 €]	[1521 € - 1601 €]	
**II Neoplasms**	1002 €	1594 €	184, 6%
***N*** **= 2938**	[959 € - 1044 €]	[1542 € - 1646 €]	
**IV Endocrine**	844 €	1575 €	18, 5%
***N*** **= 370**	[804 € - 883 €]	[1512 € - 1638 €]	
**VI nervous system**	908 €	1477 €	53, 12%
***N*** **= 427**	[882 € - 935 €]	[1441 € - 1513 €]	
**VII Diseases of the eye**	845 €		1, 0.3%
***N*** **= 344**	[838 € - 851 €]		
**VIII Diseases of the ear**	846 €		1, 1%
***N*** **= 101**	[836 € - 856 €]		
**IX Circulatory**	929 €	1439 €	387, 30%
***N*** **= 1304**	[918 € - 940 €]	[1427 € - 1427 €]	
**X respiratory**	795 €	1539 €	247, 31%
***N*** **= 792**	[759 € - 831 €]	[1512 € - 1565 €	
**XI digestive**	920 €	1517 €	124, 18%
***N*** **= 700**	[904 € - 936 €]	[1496 € - 1538 €]	
**XIII muscoskeletal**	1357 €	1537 €	36, 6%
***N*** **= 592**	[628 € - 2087 €]	[1439 € - 1636 €]	
**XIV genitourinary**	857 €	1524 €	14, 5%
***N*** **= 276**	[854 € - 861 €]	[1512 € - 1536 €]	
**XV Pregnancy**	597 €		1, 0.2%
***N*** **= 659**	[495 € - 699 €]		
**XVIII not elsewhere classified**	847 €	1531 €	22, 12%
***N*** **= 191**	[819 € - 875 €]	[1444 € - 1619 €]	
**XIX Injury, poisoning**	1002 €	1498 €	249, 18%
***N*** **= 1363**	[909 € - 1094 €]	[1451 € - 1546 €	
**XXI Factors influencing**	857 €		0, 0%
***N*** **= 116**	[854 € - 860 €]		

Note: Subgroups of patients with identical ICD-10 chapters but *N* < 100 were not analyzed. In subgroups where less than 2 patients were ventilated, costs per ventilated ICU day are not shown

## Discussion

Intensive care is a major cost component in modern healthcare systems [10]. In our sample, initiation of mechanical ventilation led to a 59% average cost increase, a very considerable increase. While costs of a ventilated ICU day differed very little between the different patient groups, the large variability of the cost *increase* associated with initiation of ventilation could open up avenues to effective resource allocation by for example focusing preventative measures, where multiple possible interventions might compete for funding, on the patient groups where avoidance of MV would be associated with the highest savings. Detailed cost data is thus useful to inform policy and optimally allocate limited resources. Our findings contribute towards this.

Overall, our results are in line with the available literature [2–5]: Dasta et al. (2005), for instance found much higher daily costs of a non-ventilated or ventilated bed day at US hospitals (US$3250 and US$4772, in 2002 values), but the relative cost increase (47%) seems comparable [2]. Other studies reported much lower extra costs of mechanical ventilation: Moran et al. (2004) determined the daily costs of a non-ventilated or ventilated bed day at Australian hospitals (AU$1616 and AU$1911, in 1991 values), corresponding to a relative cost increase of 18% [3]. The only other German study on the topic found costs of a non-ventilated or ventilated bed day of EUR 680 and EUR 946 (in 2003 values), a relative cost increase of 39% [1].

One surprising finding was that ICU patients with musculoskeletal diseases (as main diagnosis) were, on average, associated with very high daily costs even when not ventilated (€ 1357). This was especially marked when compared to ICU patients with respiratory diseases, who were associated with much lower daily costs when not ventilated (€ 795). This may imply that ICU patients with musculoskeletal diseases, on average, require higher treatment intensity even when they are not ventilated. Among the ICU patients with respiratory diseases (as main diagnosis), in contrast, the absence of ventilation might be associated with a generally lower treatment intensity.

A limitation of our study of course is the single-center nature of the data, however the sample was decently-sized and included all patients treated in the period examined, limiting some sources of bias. The competing interests of the hospital to trigger reimbursement for services rendered and the sickness funds to limit cost should result in a good level of reliability of the administrative data.

Another limitation is that daily ICU costs were not available for analysis. In practice, avoidance of one last additional day of ventilation in a given patient is expected to lead to lower cost savings than avoidance of the most expensive first day of ventilation, after which daily cost drops rapidly [2]. However, this does not detract from the usefulness of our findings on the cost differences between patient groups by the ICD-10 chapter of the main diagnosis, which has been previously underreported and is important due to the large size of the effect.

### Key points for decision makers

- Mechanical ventilation markedly increases daily ICU cost.
- The magnitude of the increase over unventilated care differs strongly between different underlying diseases.
- It might be possible to generate saving by focusing budgets for efforts to prevent necessity of ventilation on fields where initiation of ventilation would lead to a particularly pronounced cost increase.

## Conclusions

Overall, the results show substantial variability of ICU costs for patients with different underlying diseases and underline mechanical ventilation as an important driver of ICU costs. This needs to be taken into account when estimating the economic burden of diseases that require intensive care treatment with or without mechanical ventilation. More studies on the daily costs of mechanical ventilation and intensive care are duly needed.

## Supplementary information

**Additional file 1.** The cost matrix for every case. Costs are allocated to cases according to the key cost drivers for cost modules shown in the cost-matrix.

## Data Availability

The data cannot be shared since transfer of the data outside the Medical Center – University of Freiburg network is prohibited under the European Union General Data Protection Regulation (GDPR).

## Abbreviations

EUR: Euros
G-DRG: German diagnosis related groups
ICD-10: International Classification of Diseases, 10th revision
ICU: Intensive care unit
InEK: Institute for the Payment system in Hospitals
MV: Mechanical ventilation
UMCF: University Medical Center Freiburg
