# Supplementary material for: Mechanical ventilation and the daily cost of ICU care
Source: BMC Health Serv Res. 2020 Mar 31;20:267. doi: 10.1186/s12913-020-05133-5 (PMC7106643; doi:10.1186/s12913-020-05133-5)
Supplement: Supplementary file 1 — Additional file 1. The cost matrix for every case. Costs are allocated to cases according to the key cost drivers for cost modules shown in the cost-matrix. [file 12913_2020_5133_MOESM1_ESM.docx]

# Mechanical ventilation and the daily cost of ICU care

Kaier, Klaus, PhD; Heister, Thomas, PhD; Wolff, Jan, PhD; Wolkewitz, Martin, PhD

Online appendix 1: The cost matrix for every case. Costs are allocated to cases according to the key cost drivers for cost modules shown in the cost-matrix. Adapted from Vogl 2012 (1) and the InEK cost calculation handbook (2).

|  | Personnel | | | Material | | | | | Infrastructure | |
| --- | --- | --- | --- | --- | --- | --- | --- | --- | --- | --- |
| Cost category  groups  Cost-  center groups | Physicians | Nursing | Medical/ technical staff | Drugs general | Drugs individual | Implants and grafts | Material | Material individual | Medical | Non-medical |
| Ward | Care days | PPR minutes* | Care days | PPR minutes* | Actual  cost | n/a | PPR minutes* | Actual  cost | Care days | |
| Intensive care | Weighted ICU hours | | | |  | Actual  cost | Weighted ICU hours |  | ICU hours | |
| Dialysis | Weighted dialyses | | | |  | n/a | Weighted dialyses |  | Weighted dialyses | |
| Operating rooms | Setup and surgery  time | n/a | Setup and  surgery time | |  | Actual cost | Setup and surgery time |  | Setup and  surgery time | |
| Anesthesia | Anesthesia time |  | Anesthesia time | |  | n/a | Anesthesia time |  | Anesthesia time | |
| Delivery ward | Time in ward |  | Time in ward | |  |  | Time in ward |  | Time in ward | |
| Cardiac diagnostics/ therapy | Reim-bursement point system/  actual duration |  | Reimbursement point system /  actual duration | |  | Actual cost | Reim-bursement point  system/  actual duration |  | Reimbursement point system/  actual duration | |
| Endoscopic diagnostics/ therapy |  |  |  |  |  |  |  |  |  |  |
| Radiology | Reimb.  point system |  | Reimbursement point system | |  |  | Reimb.  point system |  | Reimbursement point system | |
| Laboratories |  |  |  |  |  |  |  |  |  |  |
| Further diagnostics/ therapy | Reimbursement point system/ actual duration | | | |  |  | Reimb.  point system/ duration |  | Reimbursement point system/ actual duration | |

*: PPR = Pflegepersonalregelung, staffing regulation for nursing, a standardized point system to measure nursing effort

**Bibliography**

1. Vogl M. Assessing DRG cost accounting with respect to resource allocation and tariff calculation: the case of Germany. Health Econ Rev. 2012;2(1):15.

2. DKG G. PKV (2016) Kalkulation von Behandlungskosten–Handbuch zur Anwendung in Krankenhäusern (InEK). Version 4.0. 10. Oktober. Deutsche Krankenhausverlagsgesellschaft. 2017.
